# Evaporative and Wicking Functionalities at Hot Airflows of Laser Nano-/Microstructured Ti-6Al-4V Material

**DOI:** 10.3390/nano13010218

**Published:** 2023-01-03

**Authors:** Ranran Fang, Zhonglin Pan, Jiangen Zheng, Xiaofa Wang, Rui Li, Chen Yang, Lianrui Deng, Anatoliy Y. Vorobyev

**Affiliations:** 1 School of Optoelectronic Engineering, Chongqing University of Posts and Telecommunications, 2 Chongwen Road, Nanan District, Chongqing 400065, China; 2 School of Science, Chongqing University of Posts and Telecommunications, 2 Chongwen Road, Nanan District, Chongqing 400065, China; 3 School of Automation, Chongqing University of Posts and Telecommunications, 2 Chongwen Road, Nanan District, Chongqing 400065, China

**Keywords:** femtosecond laser processing, nanostructures, microstructures, evaporative properties

## Abstract

A novel multifunctional material with efficient wicking and evaporative functionalities was fabricated using hierarchical surface nano-/microstructuring by femtosecond laser micromachining. The created material exhibits excellent multifunctional performance. Our experiments in a wind tunnel demonstrate its good wicking and evaporative functionalities under the conditions of high-temperature airflows. An important finding of this work is the significantly enhanced evaporation rate of the created material compared with the free water surface. The obtained results provide a platform for the practical implementation of Maisotsenko-cycle cooling technologies for substantially increasing efficiency in power generation, thermal management, and other evaporation-based technologies. The developed multifunctional material demonstrates long-lasting wicking and evaporative functionalities that are resistant to degradation under high-temperature airflows, indicating its suitability for practical applications.

## 1. Introduction

Burning fossil fuels (coal, oil, and natural gas) for electricity generation provides about 70% of the world’s electricity production. The combustion of these fuels produces huge amounts of environmental pollutants, such as CO_2_, SO_x_, NO_x_, and various volatile organic compounds. As a result of these and other pollutants, the climate on the Earth is changing, demonstrating a global warming trend. Recent theoretical research has revealed that applying Maisotsenko cycle (M-cycle) indirect evaporative cooling systems in power plants can increase electricity generation efficiency [1,2,3,4,5,6,7,8], thereby reducing the amount of environmental pollutants. For example, theoretical studies [7,8] have demonstrated that the application of M-cycle cooling systems in the classical Rankine power cycle enhances the efficiency of steam-turbine power generation in coal-fired power plants due to decreasing the temperature of the water supplied from the cooling tower to the condenser. Pandelidis [8] performed a numerical study of the M-cycle cooling tower and demonstrated its capability to cool the water below the inlet air wet-bulb temperature, i.e., below the limit of the traditional cooling towers. Fan et al. [9] experimentally investigated the cooling performance of the M-cycle cooling tower and proved that the M-cycle cooling can decrease the outlet water temperature below the wet bulb temperature of ambient air. Furthermore, the application of M-cycle heat and mass exchangers (MC-HMXs) in gas turbines (Brayton power cycle) substantially enhances the electricity generation efficiency and reduces the pollution emissions generated by power plants [1,2,3,4,5,6,10,11,12,13,14,15]. For example, Saghafifar and Gadalla [2] analyzed the Maisotsenko open gas turbine power cycle using a detailed air saturator model and compared it with humid air gas turbine cycles. Their results have shown that the application of the M-cycle enhances the power plant’s performance. Chen et al. [3], using finite-time thermodynamics, demonstrated that the open Maisotsenko–Brayton cycle outperforms the conventional open regenerated Brayton cycle in terms of power and efficiency performances. Numerical results of the thermodynamic assessment of applying the Maisotsenko power cycle concept in micro gas turbines have shown that this power cycle provides the highest waste heat recovery and highest power efficiency [5]. Zhu et al. [6] analyzed the bio-fueled Maisotsenko combustion turbine cycle and found an essential improvement in the exergy efficiency. Moreover, a detailed thermodynamic and economic analysis of utilizing M-cycle cooling technology in the conventional combined Brayton–Rankine power cycles demonstrated that it enhanced thermal efficiency by 6% and reduced the electricity cost by 3.8 USD/MWh [16]. Both high-temperature and low-temperature MC-HMXs are needed for enhancing efficiency in power generation. Application of the low-temperature MC-HMXs operating in a temperature range of the conventional air conditioners (<45 °C) have been recently reported in [9], indicating that some MC-HMXs developed in the field of M-cycle air-conditioning can be used for improving efficiency in power generation. The field of M-cycle air-conditioning has been in-depth discussed in a number of reviews [1,15,17,18,19]. However, the practical implementations of the high-temperature MC-HMXs remain unrealized due to the absence of multifunctional materials with efficient wicking and evaporative functionalities at high-temperature airflows, which are needed for the fabrication of wet channels. The creation of this class of materials is a challenging scientific problem that requires an extremely strong capillary action to replenish a large amount of evaporated water under hot airflow conditions. The material should also provide high interfacial evaporation efficiency. In the past, geometrically structured materials for enhancing water transport and interfacial evaporation have been actively studied for a large variety of hierarchical nano-/microscale structural geometries under still ambient air conditions [20,21,22,23,24,25]. However, a very limited number of works have studied the issues of interfacial evaporation from geometrically structured materials and their wicking performance in the presence of airflow [26,27,28]. At present, these issues remain poorly explored and understood, making the creation of materials for high-temperature M-cycle technologies a difficult task. 

Herein, we created a multifunctional material with efficient wicking and evaporative functionalities by mimicking biological materials, where these functionalities are provided through hierarchical surface nano-/microstructures [29,30]. To fabricate the hierarchical surface nano-/microstructures, we used a femtosecond laser nano-/microstructuring method that has been demonstrated to be a powerful tool in fabricating a large variety of hierarchical nano-/microstructures [31,32,33,34,35,36,37,38,39,40]. We used this method because several unique monofunctional superwicking materials have been produced previously using femtosecond laser processing, demonstrating the great potential of this method [41,42,43,44,45]. The hierarchical surface nano-/microstructure developed in our work is an array of nanostructured microgrooves fabricated on the surface of a Ti-6Al-4V alloy substrate. Our choice of Ti-6Al-4V alloy is based on a previous study that shows the long-term stability of this alloy at high temperatures [46]. The created hierarchical surface structure demonstrates an extremely strong wicking action even under airflows with a temperature of 120 °C, velocity of 14 m/s, and humidity of 1%. Our research on evaporative functionality shows that the evaporation rate of the developed material is up to seven times higher than that of a free-water surface in a pool. The created multifunctional material demonstrates long-lasting wicking and evaporative functionalities that are resistant to degradation under high-temperature airflows, indicating its suitability for practical energy-related applications.

## 2. Experimental Techniques

The femtosecond laser setup for processing the Ti-6Al-4V samples is similar to that described in detail in [46,47]. Briefly, we used a laser system Astrella (Coherent Inc., Santa Clara, CA, USA) that generates 86 fs pulses with an energy of 7.13 mJ/pulse at a maximum repetition rate of 1 kHz and with a central wavelength of 800 nm. The sample is mounted on a computer-controlled XY translation stage. A lens with a focal distance of 150 mm focuses the horizontally polarized laser beam onto the sample at a normal angle of incidence. A half-wave plate and polarizing beam-splitter cube are utilized to vary the laser power. A non-polarizing beam-splitter and power meter are used to measure the laser power. The array of the parallel microgrooves is produced by raster scanning the sample across the laser beam. The Ti-6Al-4V alloy plates were purchased from Goodfellow (Shanghai, China). Before laser processing, the received plates were cleaned in an ultrasonic cleaner with distilled water at 40 °C. In our study, the array of nanotextured microgrooves is fabricated using a laser fluence of 5.1 J/cm^2^. The step between the scanning lines is 80 μm, the pulse repetition rate is 1000 Hz, and the scanning speed is 0.9 mm/s. The laser processing was performed in air at a temperature of 23 °C and relative humidity of 50%. The dimensions of the laser-treated area were 20 × 45 mm^2^. The morphology of the fabricated wicking structure was studied using a scanning electron microscope (SEM) Sigma 300 (Zeiss, Jena, Germany) and a 3D laser scanning microscope VK-X1100 (Keyence, Itasca, IL, USA). The elemental composition of the sample surface before and after laser processing was examined using energy dispersive X-ray spectroscopy (EDS) using a Brucker XFlash 6/30 detector (Brucker, Karlsruhe, Germany). 

**Figure 1:**
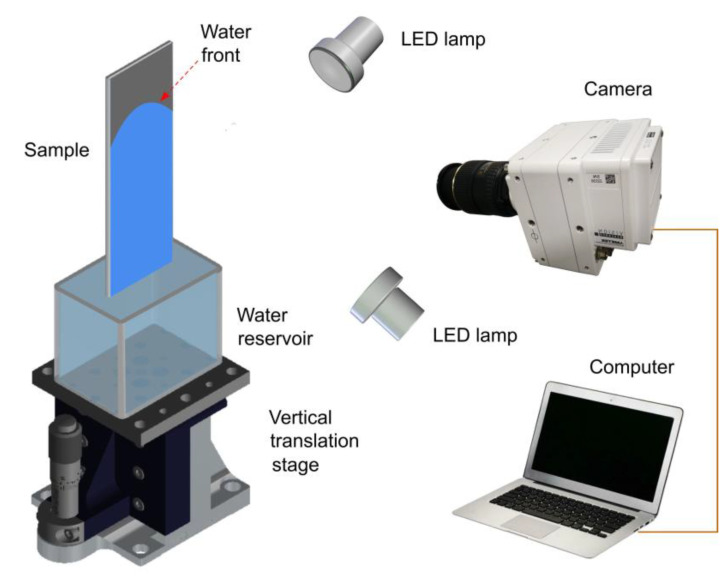
Experimental setup for high-speed video recording of capillary flow dynamics in static air at 23 °C.

**Figure 2:**
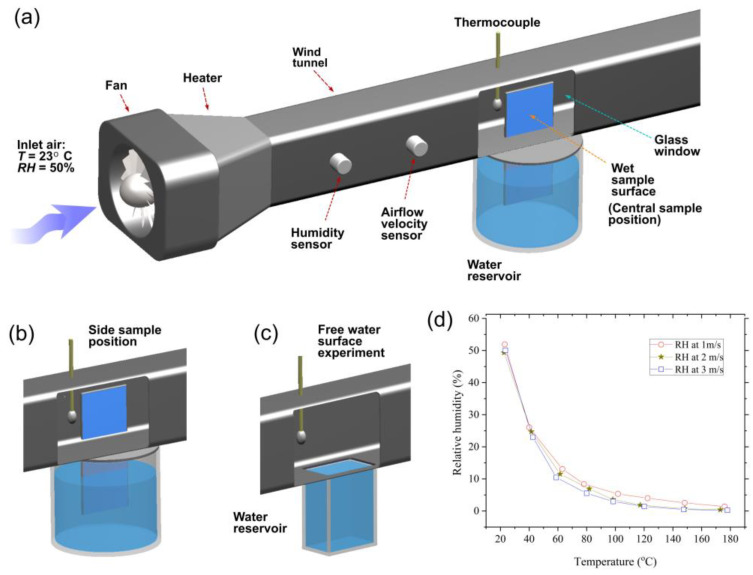
Wind tunnel setup for studying wicking and evaporative functionalities under various airflow conditions. (**a**) Wind tunnel configuration for central sample position in the wind tunnel. (**b**) Side sample position in the wind tunnel. (**c**) Wind tunnel configuration for studying the evaporation rate of free water surface. (**d**) The plot of RH as a function of temperature.

To characterize the wetting properties of the untreated and treated sample surfaces, we measure their water contact angle using an OSA 200 system (Ningbo NB Scientific Instruments, Ningbo, China). These measurements show the contact angle of 56° and ≈ 0° before and after laser processing, respectively. The wicking functionality was studied in static ambient air and under conditions of airflow. To study the wicking functionality under static air, we investigated the dynamics of water capillary flow on a vertically positioned sample using a water supply from a reservoir (Figure 1). Water spreading was captured with a high-speed VEO 710L Phantom camera (Vision Reserch Inc., Wayne, NJ, USA) at a speed of 1000 frames per second. The lens used for video recording was a Tokina AT-X M100 PRO D Macro. The sample was gently brought into contact with the water surface by translating the water reservoir vertically using a micrometer-driven linear translation stage. The experiments on the wicking functionality in the static air were performed at an ambient temperature of 23 ± 0.3 °C and relative humidity of 50 ± 2.4%. The wicking functionality under the conditions of airflow was studied using the wind tunnel setup shown in Figure 2a,b, where we measured the water rise height at various airflow velocities, temperatures, and humidities.

Evaporative functionality was studied by measuring the water evaporation rate using the wind tunnel setup shown in Figure 2. The airflow in the wind tunnel is produced by a fan. The inner cross-section dimensions of the wind tunnel are 1.25 × 2.5 cm^2^. A heater is utilized to produce hot airflows. The airflow velocity and temperature are varied using controllers. In our experiments, the airflow relative humidity changes with increasing airflow temperature. Therefore, we monitored the relative humidity (HR) in the wind tunnel using a sensor. The airflow velocity, temperature, and RH were measured by using a hot-film anemometer AR866A (SmartSensor Electronic Inc., China), thermocouple 5TC-TT-K-30-36 (Omega, Norwalk, CT, USA), and humidity sensor HMP7 (Vaisala, Helsinki, Finland), respectively. The bottom part of the sample was immersed in a water reservoir. Owing to capillary pumping of the nano-/microstructured surface, the water runs vertically uphill, wetting the entire structured surface inside the wind tunnel. The surface area of the wet sample surface exposed to airflow was 5 cm^2^ (2 × 2.5 cm^2^). The experiments were performed for two sample positions (central and side) in the wind tunnel. Figure 2a shows the central sample position, where both sides of the sample are exposed to airflow. The side sample position is demonstrated in Figure 2b, where the sample is mounted as a part of a vertical wall in the wind tunnel. In this sample location, the wet sample surface is inside the wind tunnel, whereas its back side is in the ambient laboratory air. The evaporation rate was studied under airflow velocities between 0.5 and 14.1 m/s. The range of the Reynolds numbers is 500 < *Re* < 1.8 × 10^4^, covering both laminar (*Re* < 2300), transitional (2300 < *Re* < 4000), and turbulent (*Re* > 4000) airflow regimes. The range of studied airflow temperatures is 23–120 °C, and the range of RHs is 1–50%. We find the evaporation rate *Rs* using the following equation:

(1) $$Rs=\frac{\Delta m}{At}$$

where ∆*m* is the measured mass change of the water reservoir after running the fan for a known time *t*, and *A* is the wet surface area exposed to the airflow in the wind tunnel. The measurement of *m* is performed using an analytical balance AP135W (Shimadzu, Japan) with an accuracy of 0.01 mg. For comparison, we also measured the evaporation rate *R_fs_* from a free surface of bulk water in a reservoir using the wind tunnel configuration shown in Figure 2c. In our wind tunnel experiments, the increase in airflow temperature causes RH to decrease. The plot of RH as a function of temperature is presented in Figure 2d. 

## 3. Results and Discussion

### 3.1. Characterization of Surface Structure

**Figure 3:**
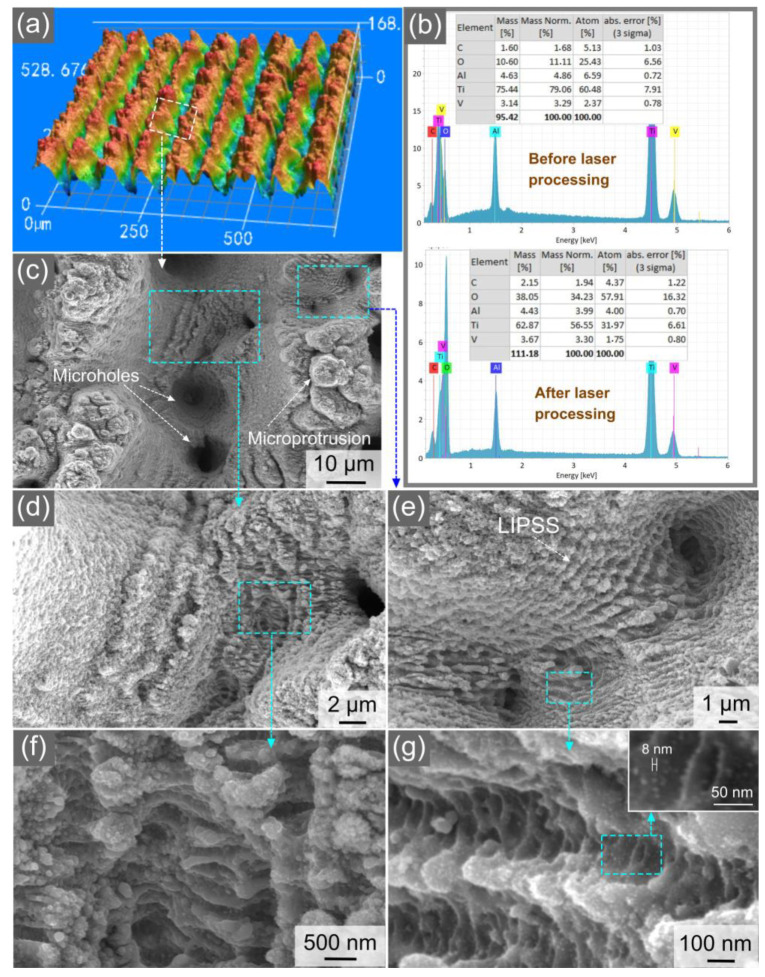
(**a**) 3D optical image of the array of parallel microgrooves on the sample surface. (**b**) Elemental composition of the sample surface before and after femtosecond laser processing. (**c**) SEM image of a microgroove. (**d**) Microstructural features on the bottom of the microgroove. (**e**) LIPSS-textured area on the microgroove ridge. (**f**) Nanostructures on the microgroove bottom. (**g**) Nanostructures on the microgroove ridge.

The 3D optical image in Figure 3a demonstrates the overall view of the produced array of parallel microgrooves. The average depth and period of the microgrooves are 76 and 80 µm, respectively. A comparison of the elemental composition of the samples before and after the femtosecond laser treatment demonstrates an essential increase in the oxygen content after femtosecond laser processing (see Figure 3b) due to the effect of femtosecond laser-induced oxidation [46,48,49]. This oxidation effect is the result of laser heating the surface of an irradiated material to a temperature substantially above the melting point, thereby activating chemical reactions between the material surface and oxygen in air. Previously, it has been found that femtosecond laser-induced oxidation of titanium results in the formation of a highly stable surface layer of nanostructured amorphous titanium dioxide (TiO_2_) [49]. Furthermore, titanium dioxide is known as a very good superhydrophilic material [50]. Therefore, the oxidation of our sample during laser processing is favorable for improving the sample performance in terms of wicking and its long-term stability. The SEM images in Figure 3c–g show the nano-/microstructural textures on the surface of the ridges and valleys of the microgrooves. The surface of the ridges, valleys, and microgroove walls are extensively textured with random fine microholes and microprotrusions in a wide variety of geometrical shapes and dimensions in a range of about 2–13 µm (Figure 3c–e). The formation of microholes is the result of the keyhole effect that usually occurs in multipulse femtosecond laser ablation at moderate and high laser fluences [41]. The surface texture of the microgrooves also includes areas of laser-induced periodic surface structures (LIPSS). The LIPSS patches are mostly produced on the bottom of the valleys and, to a lesser extent, on the ridges (Figure 3c–e). The LIPSS or surface ripples are induced through a surface plasmon polariton mechanism or some self-organization process during the laser ablation of the materials [32,40,51]. In our surface structure, the LIPSS are observed in a large variety of morphologies (see Figure 3c,e,g). The SEM images in Figure 3f,g show a wide variety of nanostructures of various geometries and dimensions. The smallest size of the nanostructures is about 9 nm (Figure 3g). Thus, the produced wicking structure is a highly hierarchical one, where the dimensions of the surface structures are in a range between 9 nm and 80 µm, covering four orders of magnitude in length scales. 

### 3.2. Characterization of Wicking Functionality

**Figure 4:**
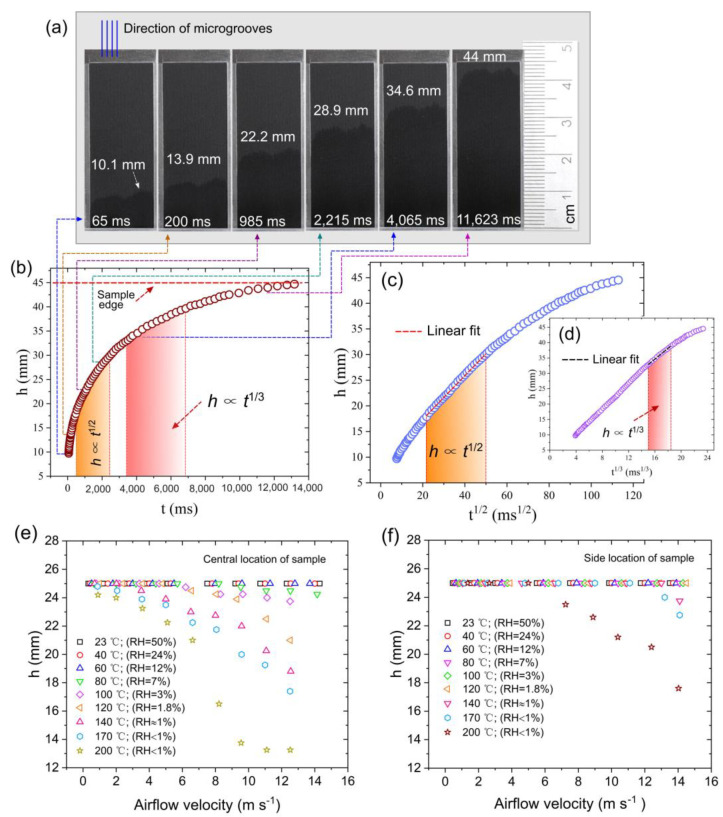
The overall dynamics of the water capillary rise on the laser-treated sample surface. (**a**) Snapshots of water rise on the surface of vertically standing sample. (**b**) Spreading distance as a function of time. (**c**) Spreading distance as a function of *t*^1/2^. (**d**) Spreading distance as a function of *t*^1/3^. (**e**) Spreading height of the wet area at central location of the sample in the wind tunnel as a function of airflow velocity at various temperatures and humidities. (**f**) Spreading height of the wet area at side location of the sample in the wind tunnel as a function of airflow velocity at various temperatures and humidities.

The wicking dynamics of a liquid in a capillary medium undergo several capillary flow regimes described by a general scaling law *h* ∝ *t^n^*, where *h* is the spreading distance of the liquid, *t* is the time, and 2 ≥ *n* ≥ 0.1 [47,52,53,54,55,56,57,58]. The most studied regime is the classical Washburn capillary flow given by the *h* ∝ *t*^1/2^ scaling law [41,42,54,55,59,60], which is observed in an extremely large variety of capillary media and considered to be a universal law of capillary flow. The Washburn *h* ∝ *t*^1/2^ dynamics relates to a steady liquid flow when the capillary force is balanced by a viscous force and the effect of gravity is negligible. The Washburn capillary flow in smooth V-microgrooves is written as follows [54]:

(2) $$h^{2}=K\left(\varphi ,\theta \right)\left[\gamma H/\mu \right]t$$

where *θ* is the contact angle, *γ* and *μ* are the surface tension and viscosity of the liquid, *H* is the groove depth, and *K*(*φ,θ*) is the geometry term, with *φ* being the groove angle. After the Washburn stage, the flow regimes follow the *h* ∝ *t^n^* scaling law with 0.5 > *n* ≥ 0.1 [56,58,61,62,63], among which the *h* ∝ *t*^1/3^ regime [56,58] is commonly observed after the termination of the Washburn flow. The *h* ∝ *t*^1/3^ dynamics in an array of V-microgrooves is given by [56] as follows:

(3) $$h\left(t\right)\approx {\left(\frac{\gamma^{2}cos^{2}\theta }{\mu \rho g}t\right)}^{1/3}$$

where *ρ* is the density of liquid and *g* is the gravitational acceleration. Besides the arrays of microgrooves [56,58], *h* ∝ *t*^1/3^ capillary flow is also observed in micropillar arrays [61] and corners [64]. The *h* ∝ *t*^1/3^ spreading dynamics occurs when the capillary force is balanced by viscous and gravitational drags [61]. The time domains of the *h* ∝ *t*^1/2^ and *h* ∝ *t*^1/3^ regimes constitute a significant part of the entire capillary flow. Therefore, we characterize the wicking performance of our sample with a focus on these capillary flow regimes. Our data on the wicking performance in static air at room temperature are presented in Figure 4a–c. As the bottom edge of the microgroove array touches the surface of the water in the reservoir, water rapidly runs vertically uphill due to capillary pumping. Figure 4a shows a sequence of water rise snapshots on the vertically positioned sample. It can be seen that the spreading distance reaches a height of 22.2 mm at 985 ms, demonstrating good wicking functionality. The spreading distance reaches 44 mm at 11,623 ms. The plot of the spreading distance as a function of time is presented in Figure 4b, where the time domains of the *h* ∝ *t*^1/2^ and *h* ∝ *t*^1/3^ capillary flow regimes are indicated. These time domains were identified by a linear fit of the *h*(*t*^1/2^) and *h*(*t*^1/3^) dependences, as shown in Figure 4c,d, respectively. The *h* ∝ *t*^1/2^ regime is observed between 478 and 2,214 ms. At the end of the *h* ∝ *t*^1/2^ regime, the water rises to a height of 28.5 mm. The time domain of the *h* ∝ *t*^1/3^ regime is identified between 3223 and 6862 ms. The water spreading distance at the end of the *h* ∝ *t*^1/3^ regime reaches 39.6 mm. Thus, there is a transition between the *h* ∝ *t*^1/2^ and *h* ∝ *t*^1/3^ regimes, which takes place between 2214 and 3223 ms. After the *h* ∝ *t*^1/3^ regime, water continues to rise and reaches the top edge of the laser-treated area at 12,743 ms (see Figure 4b). As seen from the presented data, the created material demonstrates good capillary pumping functionality.

Figure 4e,f shows the results of the wicking functionality obtained from the wind tunnel experiments under various airflow velocities, temperatures, and humidities. In these experiments, the height of the laser-treated sample area exposed to the airflow in the wind tunnel is 25 mm. The distance between the sample’s bottom edge inside the wind tunnel and the water level in the container is 10 mm. Figure 4e shows the spreading height of the wet area in the wind tunnel as a function of airflow velocity at various temperatures and humidities for the central location of the sample, where the wet side of the sample and its dry back side are both exposed to airflow (see Section 2). It can be seen that the entire laser-treated area remains wet when the airflow velocity increases from 0.5 m/s to 14 m/s in a temperature range of 23–60 °C. Due to evaporation, the spreading height of the wet area begins to decrease at *v* = 9.5 m/s and *T* = 80 °C, remaining high (24.2 mm) even at *v* = 14.2 m/s. At *T* = 140 °C, the spreading height of the wet area starts to decrease at *v* = 3.5 m/s, reducing to 18.8 mm at *v* = 12.5 m/s. As seen in Figure 4d, the wicking action is so efficient that half of the laser-treated area remains wet even at *T* = 200 °C, *v* = 12.5 m/s, and RH < 1%. At the side location of the sample (Figure 4e), the laser-treated area remains completely wet when the airflow velocity increases from 0.5 m/s to 14.1 m/s in a temperature range of 23–120 °C. The spreading height begins to descend at *T* = 140 °C and *v* ≈ 14 m/s. Figure 4e shows that the sample remains completely wet even in a velocity range of 0.5–6 m/s at *T* = 200 °C. Thus, our wind tunnel experiments demonstrate the excellent wicking performance of the created material under the conditions of high-temperature airflows.

### 3.3. Characterization of Evaporative Functionality

**Figure 5:**
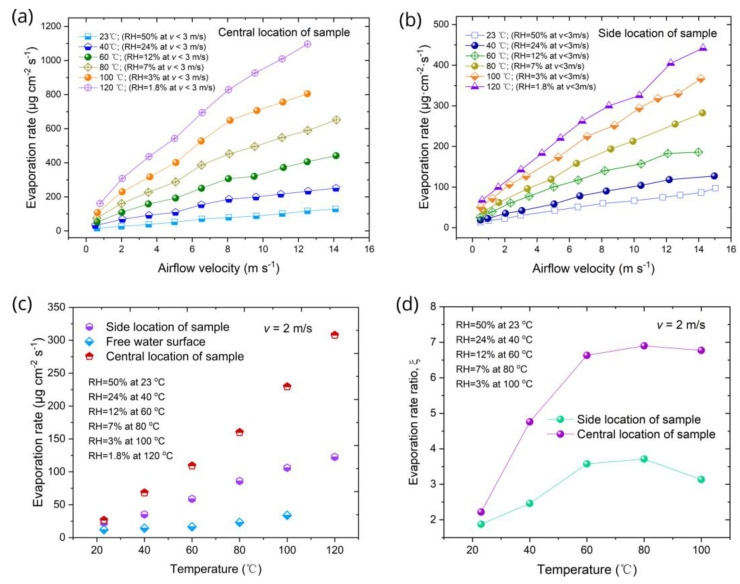
Wind tunnel experiments on wicking and evaporative functionalities under airflow conditions. (**a**) Evaporation rate *R*_s_ of the sample as a function of airflow velocity at various airflow temperatures for central sample location. (**b**) Evaporation rate *R*_s_ of the sample as a function of airflow velocity at various airflow temperatures for side sample location. (**c**) Evaporation rates of the sample and surface of the water in a container as a function of temperature at airflow velocity of 2 m/s. (**d**) Evaporation rate ratio *ξ* = *R*_s_/*R*_fs_ as a function of temperature at airflow velocity of 2 m/s.

A challenging problem in the implementation of M-cycle technologies is the creation of materials that are capable of retaining their efficient evaporative functionalities under conditions of airflow without the formation of dry-out spots. This issue is especially important in the development of high-temperature M-cycle heat and mass exchangers for power generation applications [1,2,3,4,5,6,7,8,9] where the air/gas flow temperature is above 100 °C. Here, we investigated the evaporative functionality under various airflow velocities (0.5–14.1 m/s) and temperatures (23–120 °C) using the experimental setup presented in Figure 2. The measured evaporation rates *R*_s_ as a function of airflow velocity *v* at various airflow temperatures *T* for both the central and side locations of the sample in the wind tunnel are presented in Figure 5a,b, respectively. These plots show significant enhancements in the evaporation rate with increasing *v* and *T*. The aggregated effect of temperature and humidity on the evaporation rate is demonstrated in Figure 5c,d, where the plots of *R*_s,_ *R*_fs_, and *ξ* = *R*_s_/*R*_fs_ as a function of temperature at a fixed airflow velocity of 2 m/s are shown. It is seen in Figure 5c that both *R*_s_ and *R*_fs_ essentially rise with increasing temperature and reducing RH. Our important finding is that the evaporation rate of the sample is higher than that for the free water surface by a factor of about 2–7 in the studied ranges of temperature and RH at a fixed airflow velocity of 2 m/s (Figure 5d). 

The general trends in the observed evaporation rate behaviors can be explained within the framework of classical thermodynamics. It is known that the evaporation rate *R_ev_* from a water surface is written as follows [65]:

(4) $$R_{ev}=\frac{\left(Pv_{s}-Pv_{a}\right)}{r_{BL}}$$

where *Pv_s_* is the saturated vapor pressure, *Pv_a_* is the actual vapor pressure, and *r_BL_* is the resistance of the boundary layer to the vapor flow. The water vapor pressures *Pv_s_* and *Pv_a_* are related by the equation *Pv_a_* = RH × *Pv_s_*. Therefore, the drier the air, the higher the evaporation rate. The pressure *Pv_s_* exponentially increases with temperature as follows [65,66]:

(5) $$Pv_{s}\left(T\right)=0.61e^{\frac{17.27T}{T+237.3}}$$

The resistance of the boundary layer *r*_BL_ in Equation (4) is expressed as *r_BL_* = *δ*/*D*, with *δ* being the thickness of the boundary layer at the air–water interface and $D=2.23\times 10^{-5}\left[\left(T+273.15\right)/273.15\right]\;$ being the vapor diffusion coefficient [65]. Therefore, the evaporation rate significantly increases with the increase in air temperature through *Pv*_s_(*T*) and *D*(*T*) dependences. The thickness of the boundary layer *δ* is expressed as $\delta =av_{a}{}^{-0.5}$, where *v_a_* denotes the wind velocity at the top of the boundary layer, with *a* = 3.9 and 2.26 for laminar and turbulent airflows, respectively [64]. The breaking of the bonds between the water molecules at the air–water interface is driven by the thermal energy of the water. Therefore, the hot airflow can also enhance evaporation by the heating of water at the air–water interface. As seen in Figure 5d, the evaporation rate of the created material essentially exceeds that of the free water surface. This important observation can be explained by evaporation enhancement due to water confinement within the surface nano-/microstructures of the sample [67,68]. In our experiments performed at the vertical orientation of the sample, water spreads within the microgrooves due to the formation of menisci that curve the water surface, resulting in an increase in the actual water surface area and, as a consequence, in an enhancement of the evaporation rate. At dry airflows with a high velocity and temperature, due to intensive evaporation, the water film can become confined only in the capillary fine micro- and nanostructures on the surface of the microgrooves, giving rise to other structural effects on evaporation dynamics, such as modification of interfacial thermal transport, reduction in enthalpy of evaporation, and others [20,21,22,23,24,25].

At present, the materials used in M-cycle HMXs include various types of porous polymers, fabrics, papers, ceramics, metals, and others [69,70,71,72,73]. Recently, these materials have been reviewed in [69,74]. The basic material parameter studied in the previous works is the wicking height that was investigated in still ambient air at room temperature. The study of eight wicking materials for evaporative air coolers by Pandelidis et al. [69] shows that the wicking height of the studied materials is in a range between 20 and 80 mm after 60 s. Our data in Figure 4a show that the wicking height reaches 44 mm after 11.6 s, demonstrating a very good wicking performance. Available data on the evaporation rate from the porous media, which are suitable for the comparison with our results, show that the evaporation rate of our sample is higher. For example, the evaporation rate of a porous medium composed of dispersed silica rounded particles with a diameter of 520 μm is 5.2 μg cm^2^ s (*T* = 22 °C, *v* = 0.5 m/s, and RH ≈ 10%) [75], while the evaporation rate of our sample is 13.5 μg cm^2^ s (*T* = 23 °C, *v* = 0.5 m/s, and RH ≈ 50%).

To summarize, the created material demonstrates unique wicking and enhanced evaporative functionalities under a wide range of airflow temperatures, velocities, and humidities. Furthermore, our water spreading and evaporation rate measurements repeated 6 months after laser processing of the sample exhibited negligible performance degradation of the created material, demonstrating its practical applicability. It is important to note, that advanced industrial laser systems provide currently processing rates up to 1 m^2^ s^−1^, thus enabling mass production of multifunctional metal, semiconductors, glasses, and polymer materials [32]. Although there is a general consensus that femtosecond lasers outperform other lasers in terms of nanostructuring capability and controllability [31,39,40,76], less expensive picosecond and nanosecond lasers can be also used for surface nano/microstructuring [77,78,79]. Low-cost mass production of nano/microstructured polymer and other soft materials for low-temperature HMXs can be also implemented using nanoimprinting technology, where a femtosecond laser is utilized for producing a mold and then the nano/microstructures fabricated on the mold are replicated on a soft material [80,81].

## 4. Conclusions

In this work, a multifunctional material with efficient wicking and evaporative functionalities was developed by using hierarchical surface nano-/microstructuring using a femtosecond laser. The created hierarchical nano-/microstructure spans four orders of magnitude in length scales. Due to this feature, the created material exhibits unique multifunctional performance. Our experiments in the wind tunnel demonstrated the good wicking and evaporative functionalities of the sample under conditions of airflows with high temperatures and velocities. The important finding of our work is the significantly enhanced evaporation rate of the created material compared to the free water surface. The obtained results provide a platform for the practical implementation of M-cycle technologies for increasing power generation efficiency [1,2,3,4,5,6,7,8,9], thermal management [21,23,24], and other evaporation-based technologies [82,83]. The developed multifunctional material demonstrates long-lasting wicking and evaporative functionalities that are resistant to degradation under high-temperature airflows, making it suitable for practical applications.

## Data Availability

Data are contained within the article.
